# Impact of a Mindfulness-Based Intervention on Symptoms and Emotion Regulation Strategies in Young Adolescents From the General Population: A Randomized Controlled Trial

**DOI:** 10.1155/da/2679049

**Published:** 2025-06-10

**Authors:** Camille Piguet, Zeynep Celen, Ben Meuleman, Zoé Schilliger, Mariana Magnus Smith, Erik Mendola, Eleonore Pham, Sondes Jouabli, Vladimira Ivanova, Ryan J. Murray, Paul Klauser, Arnaud Merglen

**Affiliations:** ^1^General Pediatrics Division, Women, Child and Adolescent Department, Geneva University Hospitals and Faculty of Medicine, University of Geneva, Geneva, Switzerland; ^2^Psychiatric Department, Faculty of Medicine, University of Geneva, Geneva, Switzerland; ^3^Swiss Center for Affective Sciences, University of Geneva, Geneva, Switzerland; ^4^Center for Psychiatric Neuroscience, Department of Psychiatry, Lausanne University Hospital and the University of Lausanne, Lausanne, Switzerland; ^5^Service of Child and Adolescent Psychiatry, Department of Psychiatry, Lausanne University Hospital and the University of Lausanne, Lausanne, Switzerland

**Keywords:** adolescents, emotion regulation, internalized symptoms, mindfulness, randomized controlled trial

## Abstract

Adolescence is marked by major puberty-induced changes including increased reactivity to stress and a peak incidence of mental disorders. The implementation of early interventions during this developmental period is essential to prevent mental disorders. In this clinical trial, we examined the effects of a mindfulness-based intervention (MBI) on internalizing symptoms, affects, and emotion-regulation strategies in a nonclinical sample of young adolescents. Seventy adolescents (41 girls) from the general population, aged between 13 and 15 years, were enrolled in a randomized controlled trial (RCT) that compared an 8-week MBI designed for adolescents and a waiting list. Levels of stress, anxiety and depressive symptoms, positive and negative affects, as well as emotion regulation strategies were measured before and after the intervention (4.8 weeks ± 4 SD) using self-reported questionnaires. We found no effect of our MBI on all self-reported measures, including stress, anxiety, depression, and positive and negative affects, as well as an emotion regulation strategies. Trait mindfulness was negatively correlated with measures of stress, anxiety, and negative affects. The intervention was very well accepted (only one dropout) with a high degree of satisfaction among participants. Individual responses to the intervention were very heterogeneous. Mindfulness practice in non-help-seeking adolescents was very well received, but did not show any benefit on symptoms, affects, or emotion regulation. This is consistent with the literature suggesting a better response in clinical than in nonclinical samples. Longer-term effects remain to be investigated, as does the possibility of identifying individuals who respond best to this early intervention.

**Trial Registration:** ClinicalTrials.gov identifier: NCT04711694

## 1. Introduction

Adolescence is a developmental period extending from puberty to adulthood and during which many neurobehavioral changes take place [1]. The structure of the brain undergoes further maturation associated with shaping of emotional coping skills and stress response [2, 3]. As prefrontal regions mature, more cognitive control arises over limbic-related emotional reactivity [4, 5]. These changes lead to heightened sensitivity to stress, which in turn increases vulnerability to psychiatric disorders [6–8], with a peak age of onset at 14.5 [9]. Emotion dysregulation, defined as a state where emotion regulation processes are not able to influence the experienced emotion and resulting behavior [10], is both a normal feature of early adolescence [11] and a common feature in adult mental disorders [12–14]. Therefore, the root of maladaptive emotion regulation could take place during this period of cortico-striato-thalamo-cortical network structuration which develops during adolescence and seems especially stress-sensitive [4, 5]. Stress sensitivity and maladaptive emotion regulation represent vulnerability traits, especially for mood disorders [15] and emotion dysregulation disorders such as borderline personality disorder and ADHD [16, 17].

Emerging psychiatric disorders can manifest during adolescence with subclinical and aspecific symptoms. Thus, interest is growing towards a stage-oriented classification rather than the widely used disease-specific one [18–20]. Despite a period of heightened sensitivity for mental disorders, adolescence is also seen as a critical window of opportunity, hence, the growing interest in early interventions that can target vulnerability factors transdiagnostically, focusing on young people's current needs [21]. Research is, therefore, needed to assess the effectiveness and ethicality of these early interventions [22]. Early interventions are actually very close to the concept of resiliency: one's ability to remain healthy while facing adversity, focusing more on the protective mechanisms to improve treatment and prevention [23]. Resilience is associated with mental health in children and adolescents, but further research is needed on the underlying mechanisms of protection [24, 25]. Mindfulness-based interventions (MBIs) might be one approach targeting these stress coping competencies much needed by youth [26].

Mindfulness meditation is historically linked with eastern traditions such as Buddhism; however, its practice can now be secular and manualized [27]. This and the growing demand for nonpharmacological treatments could explain the growing interest aroused by MBI. Mindfulness can be defined as “The awareness that arises from paying attention, on purpose, in the present moment, with a nonjudgmental attitude” [28]. Individuals can naturally have this trait at various degrees and it can be enhanced by meditation practice. After being initially designed for stress reduction (MBSR) and prevention of depression relapses (mindfulness based cognitive therapy (MBCT)), MBIs are now used in various contexts and ages in both clinical and nonclinical populations [29, 30]. Its efficacy is uncontested on many psychosocial conditions and well-being in different healthcare settings, even if further research is required to clarify the cost effectiveness, the usefulness for specific populations [31], and the means through which it helps [32]. The general improvement in psychological distress seems to be robust [33], as well as the specific impact on anxiety disorders in adults, where it was shown noninferior to antidepressant SSRI [34].

In adolescents, latest studies suggest that mindfulness can be implemented to improve physical and mental health [35] and that it can be performed in various outpatient settings [36]. Reviews of preliminary results in youth were promising, albeit moderate, and invited further research [37]. For example, a meta-analysis of 20 randomized controlled trials (RCTs) evaluating the impact of mindfulness on children and adolescents younger than 18 years old failed to find a specific effect on anxiety for adolescents. They used random effects meta-regressions to evaluate the impact of age, intervention setting, control type, research location, and intervention dosage on response. The effects were overall weak, if a bit more promising in younger children compared to adolescents [38]. On the other hand, a larger recent meta-analysis regrouping 66 RCTs on MBI in children and adolescents in various settings confirmed the positive effect of mindfulness on stress and anxiety, even against an active control [39]. However, the impact on attention and cognitive function is more difficult to capture. The effect size seems larger in clinical populations [37, 40]. Some mixed findings are emerging, for example, no effect on psychological distress in younger adolescents [41] or no effect on mental health in school settings [42], suggesting that MBIs for adolescents are not a one-size-fits-all approach. Therefore, while a positive effect of mindfulness trained self-regulation on various outcomes related to key health behavior has been documented in adults, more research is needed for younger participants and for understanding of the underlying psychological processes [43].

Our aim was to measure the impact of MBI on young adolescents between 13 and 15 years old, just before the peak of incidence of psychiatric disorders, but also the period where emotion regulation is developmentally at its lowest. We kept the age range narrow in order to minimize confounding factors such as hormonal and physical development. We chose to recruit participants from the general population, interested by meditation practice, to test the impact of MBI on various subjective dimensions of emotional well-being, in the context of a larger study on neurobiological mechanisms of mindfulness meditation. The neuroimaging and biological outcomes of this study are reported elsewhere. We report here the results from the subjective reports. We hypothesized that MBI would reduce self-reported levels of anxiety, stress, and depressive symptoms, increase positive affects, as well as adaptive emotion regulation strategies, compared to waiting list. We also hypothesized that the impact of MBI would be influenced by the level of baseline anxiety or history of depressive episode.

## 2. Methods

### 2.1. Sample and Design

**Figure 1:**
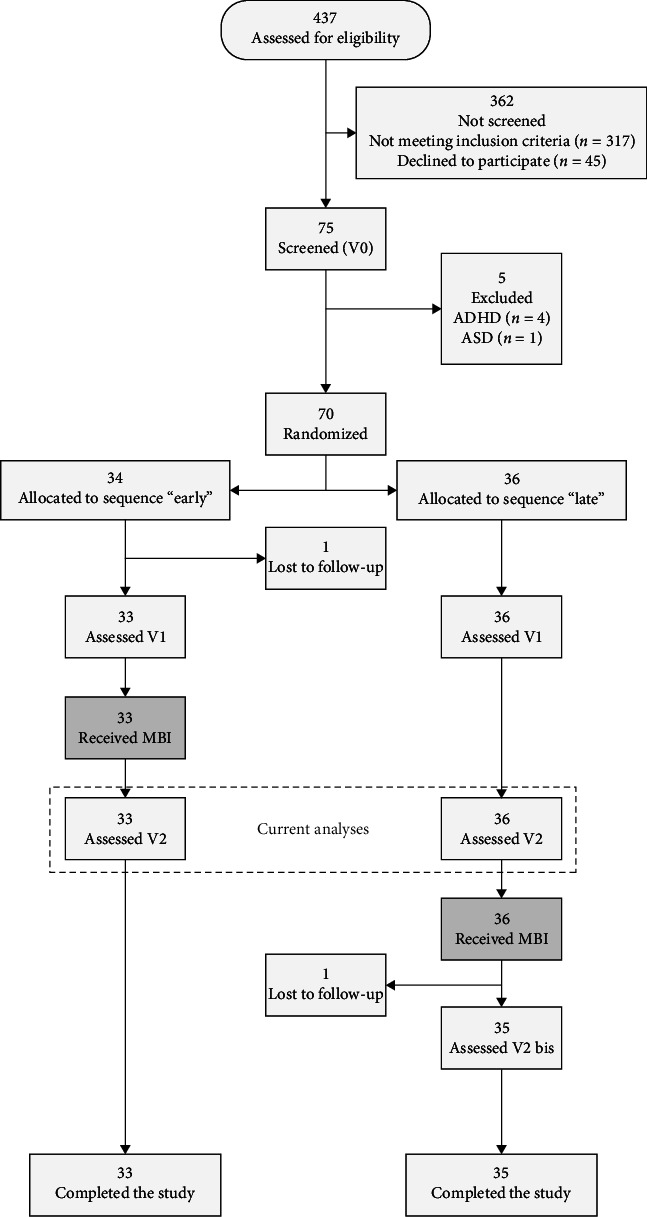
CONSORT flow diagram. ADHD, attention-deficit/hyperactivity disorder; ASD, autism spectrum disorder; MBI, mindfulness-based intervention.

The Mindfulteen Study is an experimental RCT targeting two types of outcomes: (1) neurobiological outcomes to reveal neural mechanisms involved in mindfulness; (2) clinical outcomes that explore internalizing symptoms, affects, stress coping, and emotion regulation. The initial sample target was 120 participants and the reporting follows CONSORT guidelines, including publication of the study protocol (for further details, please refer to Piguet et al. [44] and Supporting Information 1). However, due to time constraints and the impact of the COVID-19 pandemic (inclusion from February 2019 to December 2021), we were unable to reach our target. Before randomization, participants were stratified according to their trait score on the State–Trait Anxiety Inventory for Children (STAI-C): low anxiety group (≤31) or high anxiety group (>31), based on a nonpublished median in a similar community sample of schoolchildren (*n* = 535; 10–15 years old) [44]. After inclusion and assignation to high or low-anxiety group, participants in each group were randomized in these two anxiety strata, using an internet randomization service (https://www.sealedenvelope.com/) between early MBI group and waiting list (i.e., control group). The study coordinator enrolled the participants (MMS), then, the psychologists conducted the inclusion visit (Eleonore Pham, Vladimira Ivanova, and Sondes Jouabli) and finally the study coordinator attributed the group using seal envelope. Research assistants collecting the data and statistician analyzing the data were blind to the group assignment. The Mindfulteen Study was conducted in Geneva, Switzerland, and recruited 70 nonclinical adolescents between 13 and 15 years old, through a comprehensive communication strategy which included social media, hospitals, pediatricians, schools, and a website. This resulted in 437 initial contacts (see CONSORT diagram in Figure 1). Exclusion criteria were any psychiatric conditions except for current anxiety disorders without comorbidities and/or past depressive episode resolved for at least 6 months, as assessed by K-SADS interview (see below), current psychotherapy or other co-intervention (defined as more than six visits with a mental health professional in the previous 6 months), claustrophobia and pregnancy, inability to participate to groups [44]. Mindfulteen Study protocol was approved by the Geneva Regional Research Ethical Committee on January 9, 2019 (CCER 2018-01731). All participants and their legal representative sign the informed consent forms before the first visit. Participants were compensated for each research visit with remuneration ranging from 15 to 62 US$. The amount was contingent upon the nature of the visit, with questionnaires and interviews warranting a lower reimbursement than visits involving more invasive procedures, such as blood sampling and magnetic resonance imaging (MRI). These payments were made in accordance with local regulations. However, no compensation was provided for participation in the group meditation intervention. There is no ethical concern nor major risk associated with this study. Participants were expected to benefit from the MBI intervention and have been closely monitored by trained psychologists and physicians to manage any stress or anxiety that would be associated with the procedures

### 2.2. Procedure

Assessments were performed before intervention and after intervention or waiting period (mean 4.8 weeks, SD 4 weeks, range: 3–169 days). The intervention consisted of an 8-week MBI training, followed by mandatory 4 weeks of weekly booster sessions. Each weekly group session (up to 12 participants) lasted 90 min. Participants were also encouraged to practice individually daily with the help of a smartphone app, designed for the study. The proposed MBI was an adolescent age-adapted MBCT. MMS and AM, both certified seasoned instructors in MBSR and MBCT for adolescent populations developed and adapted a previous version of the program [45] for this study. Finally, AM and MMS conducted the groups assisted by other team members. A detailed description of the intervention has been already published [44]. After completion of the intervention all participants retained access to the smartphone app, supporting a possible transition to a sustained practice.

The program was intended as in person groups of 8–12 adolescents with two trained instructors. However, when COVID-19 pandemic forced us to stop meeting in groups, we decided to continue using video-conferencing. Finally, only one out of the eight groups used video conferencing (five participants) and this was taken into account during the analyses.

### 2.3. Questionnaires

All the clinical variables were coded into a standardized database using Teleform semiautomated program and each data entry was double checked by the study coordinator. For screening (V0), participants underwent a structured K-SADS interview [46] with a trained clinical psychologist and the Spielberger STAI-C for stratification [47, 48]. The K-SADS was used to determine puberty status (yes/no definition question). Upon inclusion, self-reported questionnaires were filled by the participants, in two different sessions: complementary measures of anxiety and depression (Multidimensional Anxiety Scale For Children (MASC) [49], Depression Anxiety Stress Scale (DASS) [50], and Beck Depression Inventory [51]); general functioning (Strength and Difficulties Questionnaire (S&D) [52]) and WHO-5 well-being index; trait mindfulness (CAMM [53]); and complementary measures of emotion regulation: Positive and Negative Affects Scale (PANAS [54]); Emotion Regulation Questionnaire, subscales for cognitive reappraisal (CR) and emotion suppression (ES) [55]; Emotion Awareness Questionnaire, subscale for bodily awareness (BA), and differentiation of emotions “Diff emotions” [56]. Here we report on these 13 outcomes measures, pre- (V0 and V1) and postintervention (V2) or waiting period. Participants also filled an in-house satisfaction questionnaire, with eight semiopen questions: (1) How would you rate this program (0–10)? (2) What did you learn (if anything)? (3) Which practices did you enjoy the most? (4) Which practices were the most difficult for you? (5) Would you recommend this program to a friend? (6) Anything you would suggest changing? (7) Have you used MindApp? How did you find it? (8) Any other comments?

### 2.4. Data Analysis

Data analysis proceeded in three steps (a) preprocessing, (b) descriptive analysis, and (c) inferential modeling.

Preprocessing. In the preprocessing step, manually transcribed data were cross-checked against the automatic transcription system and corrected where necessary. The cross-check revealed that questionnaires contained only 0.33% manual errors on average and maximally 1.70% (of all values). Following the cross-check, questionnaires were aggregated into relevant subscale and total scale scores. In case of missing items, these scores were adjusted for the missing item. Overall levels of missing data were extremely low, however, at 1.5% of all data. Finally, the data were converted from so-called “wide format” to “long format” for analysis, with two rows of data per participant, corresponding to the screening visit (V0) and V2 visits (postintervention).

Descriptive analysis. In the descriptive analysis step, we tabulated means and standard errors for all variables, both for the whole sample and per group (MBI and waiting), separately. For categorical variables, cross-frequencies were tabulated instead of means. Finally, we calculated Pearson correlations between all variables, after decorrelating values for within-subject and within-time correlation. 1

Inferential modeling. In the inferential modeling step, we conducted multilevel ANOVAs of group (MBI and waiting) × visit (V0 and V2) on each of the 13 outcome measures. This analysis was repeated for three subsets: (1) the complete sample ( = intention-to-treat analysis), (2) excluding participants in both groups who had the possibility to be randomized to a video conferencing intervention, and (3) excluding MBI participants who received the intervention on video conferencing ( = per-protocol analysis). These three subsets contrasted results between the original design, as intended, and those at risk or having received an intervention protocol that differed from what was intended.

Multilevel models contained each one of the 13 outcome measures as dependent variable, interacting group, and visit variables as fixed independent variables and a random intercept for subjects to account for repeated measures correlation. Although inferential tests under this model are identical to the ones obtained for the equivalent repeated measures (*M*) ANOVA, we opted for the multilevel approach because it facilitated residual diagnostics and because it enabled the inclusion of subject- and visit-varying covariates simultaneously, if necessary. For each fitted model, an *F*-test was calculated for the group × visit interaction and *t*-tests for pairwise comparisons between visits within groups, with Bayes factors as a measure of effect size. All inferential tests were conducted at a reduced significance level of *α* = 0.005, following recommendations to reduce false positive findings in social sciences [57]. Residual diagnostics were conducted on each fitted model to check against influential cases (Cook's distance), nonnormal residuals, and nonconstant variance. 2

Finally, several control analyses were conducted to exclude the influence of important moderating and confounding effects on the intervention for the 13 outcome variables. Moderators included baseline anxiety stratum, anxiety disorder, and past depression, for which we fitted a three-way multilevel ANOVA of group × visit × moderator. For anxiety stratum, STAIT was excluded from analysis due to the stratum itself having been derived from baseline STAIT. Confounders included continuous age in months at visit, as a pure effect of time passing, and categorical visit month, as a seasonal influence. For confounders we refitted the group × visit multilevel ANOVAs with the confounders as controlling covariates.

All analyses were conducted using the R statistical software [58], with packages lme4 and lmerTest for multilevel modeling [59, 60], DHARMa for residual diagnostics [61], brms and bayestestR for Bayes factors [62, 63], and corrplot and visreg for data visualization [64, 65].

## 3. Results

### 3.1. Descriptive analysis

We recruited 70 adolescents (41 girls), with a mean age at baseline of 14.3 years. One adolescent dropped out the protocol before starting the intervention. Out of 70 participants, 55 (78.6%) had no lifetime history of psychiatric disorders, while 15 reported previous depressive episodes or current anxiety disorders (Table 1).

The two groups did not differ significantly in terms of demographical characteristics, including mean age, mean duration between V0 and V2, sex, puberty onset, or presence of a generalized anxiety disorder. The two groups also did not differ significantly in terms of COVID-19 context or video conference participation in the intervention. Finally, the two groups did not differ significantly in mean value for the 13 outcome measures at baseline, with similar balance of low- and high-anxious participants (see STAIT strata in Table 2).

Overall, the program was well attended (10.85 sessions/12 (SD 0.25)), with 8.7/10 satisfaction rate and 90% of participants that would recommend the program to a friend. At the open question “what did you learn (if anything) with this group,” 44% of adolescents answered with themes related to emotion regulation, coping with stress; 20% on the theme of being less judgmental with oneself; 18% on the theme of knowing oneself better, and 18% other themes.

### 3.2. Inferential Modeling

**Figure 2:**
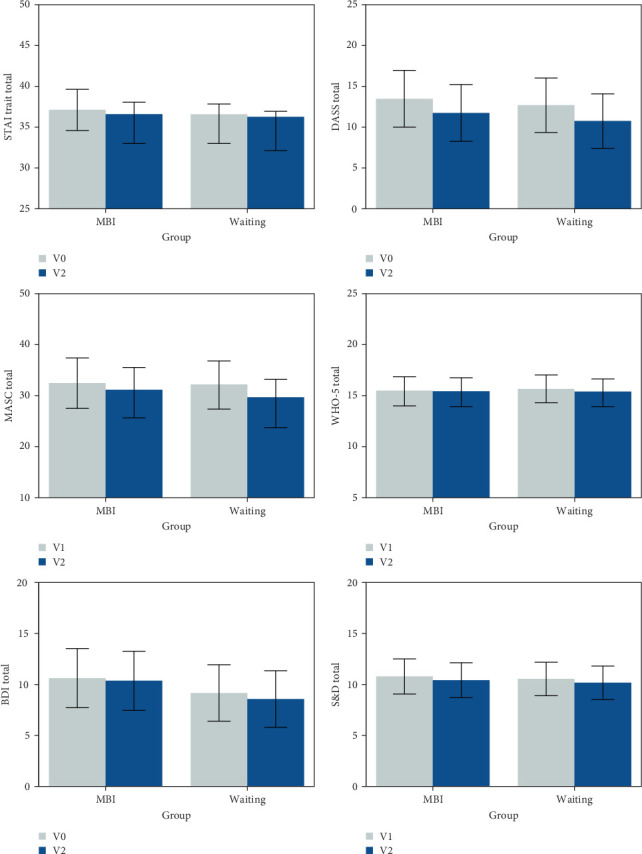
Group × visit estimated means and standard errors for Spielberger State Trait Anxiety Inventory-Trait (STAI trait total), Depression Anxiety Stress Scale (DASS total), Multidimensional Anxiety Scale for Children (MASC total), WHO-5 well-being Inventory, Beck Depression Inventory (BDI), and Strength and Difficulties Questionnaire total score (S&D total). See Supporting Information 2: Figure S1 for graphs of additional outcome variables.

Multilevel ANOVAs on the 13 outcome measures for all participants did not reveal any significant group × visit interactions (all *p*  > 0.1, all Bayes factors <1), including for the primary anxiety measure, STAIT, *F* (1.69) = 0.239, *p*=0.6263, and BF = 0.205. Planned comparisons of visits within groups also did not reveal any significant effects, except for a trend to a significant reduction in MASC Total for the waiting group, V0–V2 = 3.61, 95%CI [0.43,6.79], *t* (69) = 2.27, *p*=0.0265, and BF = 1.34 (Table 3 and Figure2). The multilevel ANOVAs additionally revealed two trends toward a main effect for visit, such that DASS Total reduced in both groups, V0–V2 = 1.84, 95%CI [0.21,3.46], *t* (69) = 2.25, *p*=0.0275, and BF = 1.17; and MASC Total reduced in both groups, V0–V2 = 2.74, 95%CI [0.44,5.04], *t* (69) = 2.38, *p*=0.0201, and BF = 1.22. We show graphically here the results for STAIT, DASS, MASC, BDI, S&D, and WHO-5 well-being. Additional representation of nonsignificant results (CAMM total score and PANAS, ERQ, and EAQ subscores) can be found in Supporting Information 2: Figure S1.

Multilevel ANOVAs excluding participants that had the chance to be randomized to an online (video conference) intervention also did not reveal significant group × visit interactions (all *p* > 0.1, all Bayes factors < 1). Planned comparisons of visits within groups yielded the identical pattern of results as for the whole sample. The multilevel ANOVAs additionally revealed two trends toward a main effect for visit, such that MASC Total reduced in both groups, V0–V2 = 2.80, 95%CI [0.33,5.28], *t* (50) = 2.29, *p*=0.0270, and BF = 1.35; and EAQ differentiating emotions increased in both groups, V0–V2 = −0.84, 95%CI [−1.57,−0.11], *t* (50) = −2.30, *p*=0.0257, and BF = 1.29 (Supporting Information 2: Table S1).

Multilevel ANOVAs excluding participants who actually received their intervention on video conference also did not reveal significant group × visit interactions (all *p*  > 0.1, all Bayes factors < 1). Planned comparisons of visits within groups yielded the identical pattern of results as for the whole sample, with an additional trend to a significant increase in ERQ ES for the MBI group, V0–V2 = −1.61, 95%CI [−2.97,−0.25], *t* (64) = 2.36, *p*=0.0214, and BF = 2.00. The multilevel ANOVAs additionally revealed one trend toward a main effect for visit, such that MASC Total reduced in both groups, V0–V2 = 2.54, 95%CI [0.15,4.92], *t* (57) = 2.13, *p*=0.0377, and BF = 1.08 (Supporting Information 2: Table S2).

Residual diagnostics of models revealed no issues with nonnormality. For all except one outcome (ERQ CR), influential cases were identified that exceeded the critical *F*-threshold of Cook's distance. However, rerunning the multilevel ANOVAs with these cases excluded did not produce significant group × visit interaction effects.

Finally, secondary multilevel ANOVAs to exclude moderating and confounding influences revealed no influence of anxiety stratum, generalized anxiety disorder, age in months, or visit month, on any of the 13 outcomes. By contrast, the analysis revealed five significant moderation effects of depressive episodes on STAIT, DASS total, BDI, S&D, and EAQ-differentiating emotions. Among adolescents who had had at least one depressive episode in the past (*N* = 8), participants in the MBI group (*N* = 4) significantly reduced STAIT and DASS total, and significantly increased EAQ-differentiating emotions versus the waiting group (Supporting Information 2: Table S3).

## 4. Discussion

We conducted a RCT to investigate the short-term impact of a MBI specifically designed for teenagers in a nonclinical sample of young adolescents from the general population and we report here findings from 13 self-reported questionnaires measuring the levels of stress, internalizing symptoms, and positive and negative affects as well as emotion regulation strategies.

Overall, participants were very well engaged in the study, with a high satisfaction rate and only one single dropout. However, we found no effect of MBI on the levels of stress, anxiety, depression, and positive and negative affects as well as regarding emotion regulation strategies. We did find a moderator, as adolescents with past depressive episode showed significant response to mindfulness intervention. Overall, the response was very heterogeneous. The lack of short-term effect of our intervention might be put in perspective of a recent large RCT well conducted in schools (i.e., MYRIAD study), in the same age-range population, that has also reported no benefit of MBI in promoting mental health compared to socioemotional skills teaching [42]. On the other hand, a recent meta-analysis of 66 RCT on MBIs in people younger than 19 years old confirmed a positive impact of MBI on anxiety, although the conclusions are more cautious than in the previous version of the meta-analysis [39]. However, many studies so far in the mindfulness literature have limitations, such as nonexperimental design, allegiance bias, and heterogeneity in interventions as well as outcomes and publication bias, that might have led to overestimation of the expected results [66].

Here, we do find improvement on most of the clinical scale, including all scales related to anxiety, but irrespective of the intervention group. This is consistent with expected improvement in emotion regulation strategies in adolescents of this age group leading to a decrease in anxiety over time in healthy adolescent population [67–69].

Several hypotheses can be made regarding the negative findings of our RCT. One is that the questionnaires used did not capture the subjective dimensions impacted by the MBI since participants in our qualitative studies did report spontaneously positive effects [70]. Another hypothesis is that the questionnaires captured only short-lived changes and in particular that while interoception and emotion awareness improve with mindfulness programs, so does the consciousness of anxiety. A similar effect is seen for the trait mindfulness dimension itself, where a decrease in trait mindfulness, as reported by the participants, is often qualitatively reported after 8-weeks programs, after participants realize what this concept actually encompasses. One study did show an effect on depression and anxiety scales in adolescents after 4 months, but not directly after the program itself [71]. In the same direction, very short interventions have shown nonsignificant results, but trends in ADHD adolescents [72]. Essentially, in some participants, short-term effects might result in a temporary decrease in trait mindfulness and an increase in anxiety as measured by questionnaires. The question of dose-dependent effect in mindfulness research is well-known and even more for adolescents where the programs are made “lighter” and might therefore be diluted [73]. Therefore, the questions of the duration of the intervention and how long-term results last should be central to mindfulness research.

Another question is whether MBI might sometimes trigger additional negative side effects, including anxiety. No formal monitoring of adverse effects was conducted, and the course of anxiety and negative events occurring during the study were very variable. Clinical research reporting the potential negative aspects of meditation is scarce but growing, with up to 25% of adult meditators experiencing some level of unpleasant psychological experience [74]. In another study, while participants were all satisfied with the intervention, up to 10.4% reported negative effects lasting more than 1 month including anxiety [75], hence, the necessity to understand how much and in which time frame mindfulness shows its positive effect [76]. For example, a large study with older participants showed that while they reported more subjective stress after mindfulness and yoga training, they also coped better with stress and presented with diminished volume of amygdala [77]. Therefore, while potential side effects are still minimal compared to antidepressants for example [78], understanding the timing of response to mindfulness, the underlying mechanisms and the settings in which non-clinical MBIs are useful warrants further research [79].

Another important point raised by these results is the heterogeneity of the changes. Some participants did report a decrease in symptoms' levels, while others reported an increase. Clinically, we can also report that the time spent practicing at home was also very heterogeneous, and adherence to treatment is commonly low in teenagers [80]. Our hypothesis that the participants presenting more severe anxiety levels in the beginning would benefit more from MBI, did not hold true in this sample. Suffering from an anxiety disorder did not moderate the relationship between group and anxiety levels. We did find that the intervention effect was moderated by past depressive episode in a little subgroup of our sample. While we cannot draw robust conclusions from this exploratory analysis, this is consistent with the well-described effect of mindfulness on prevention of depressive relapses [81]. This is also consistent with reports in the literature tending to advocate for a stronger effect in clinical samples [37]. Despite the COVID-19 pandemic starting in the middle of the study (i.e., March 2020) and the fact that some times of year can be more stressful than others (e.g., school exams), we found no effect of time of the year on any of the 13 outcomes. This is surprising given the global increase in stress and anxiety reported in the literature [82], but this might concern adolescents that are older or have other vulnerability factors. A few studies also suggest that mindfulness may have increased resilience among adolescents during the COVID-19 pandemic [83, 84]. Finally, we explored if online sessions, that were necessary for one group, could mediate some of the results, but found no effect.

More importantly, several similar negative findings have emerged in the literature since we started our RCT, evaluating the impact of MBIs in nonclinical settings and in particular universal interventions in school settings. These negative well conducted RCTs [39, 42, 85, 86] seem to point toward two important points: participation in MBI should be voluntary and motivating for young people, which was the case in our study to the extent of being part of a neuroscience experiment. And the developmental age is very important, with different studies pointing toward the idea that early adolescence might be the moment with the most barriers to the impact of mindfulness training, given the heightened emotion dysregulation, and the lack of cognitive resources necessary to fully beneficiate from the core component of mindfulness intervention [66]. In adult subjects, a recent meta-analysis showed that people with higher level of psychological symptoms were more likely to present some degree of decreased mental health after MBI, but participants with interpersonal problems were more likely to experience benefits of meditation [87]. These types of findings need replication and specific research in younger populations. Recent meta-analysis of the impact of mindfulness in nonclinical samples of adults was able to report a main effect on psychological distress, but failed to find specific moderator of the response [79]. To identify individual predictors of response, individual participant data meta-analyses seem to be necessary given the small to medium effect of mindfulness [88].

Limitations: The target sample was not reached due to the constraint of time and funding in the pandemic period. Therefore, the limited size of the cohort could also explain the non-significant results. Effect sizes of MBIs in adolescents tend to be small [39]. Therefore, large samples are required. However, it can be argued that our design was rigorous, and the nonsignificant results, despite a high satisfaction rate, provide an opportunity for reflection on the heterogeneity in response and the instruments used in mindfulness research. Finally, the lack of long-term longitudinal data is also a major limitation of our study, since, we cannot rule out the hypothesis that effects of mindfulness are slow to appear.

In conclusion, MBI in non-help-seeking young adolescents from the general population has been shown to truly engage participants according to core mindfulness principles and without any reported side effects. However, we found no additional benefit of MBI at the group level, in comparison to a “waiting list” in any of the self-rated measures related to stress, anxiety, depression, affects, or emotion regulation. Participants with previous depressive episode did show a positive effect of MBI; nevertheless, there was a high level of response heterogeneity among participants and the moderate sample size strongly limited additional sub-groups analyses that would have allowed to highlight the characteristics of the adolescents who best responded to MBI. Finally, the absence of short-term effects should not mask the possibility of longer-term benefits of mindfulness.

## Figures and Tables

**Table 1:** Demographical characteristics.

Variable	Mean (SE)			Range
	All (70)	MBI (33)	Waiting (36)	
Baseline age (years)	14.30 (0.11)	14.29 (0.15)	14.3 (0.15)	13.07–16.01
Duration total (days)	191.28 (9.74)	123.88 (6.54)	251.19 (9.57)	78–394
Duration V0–V2 (days)	128.19 (4.83)	123.88 (6.54)	132.03 (7.06)	61–256
Mindfulness sessions (out of 8)	—	7.48 (0.15)	—	—
Mindfulness sessions (out of 12)	—	10.85(0.25)	—	—
Sex (F/M)	41/29	22/11	18/18	.
Puberty (no/yes)	20/47	7/23	13/23	.
Difficulties in KSADS (no/yes)	56/13	29/4	27/9	.
Panic attacks (no/yes)	60/10	26/7	33/3	.
Social phobia (no/yes)	63/7	28/5	34/2	.
GAD (no/yes)	61/9	27/6	33/3	.
STAIT strata (LA/HA)	20/50	8/25	12/24	.
Video conference (no/yes)	57/12	28/5	29/7	.
COVID-19 (before/during)	22/48	8/25	14/22	.

*Note*: Total *N* = 70, if less means missing value.

**Table 2:** Means and standard errors of outcome measures at baseline (V0/V1).

Variable (V0/V1)	Mean (SE)		Waiting (36)	Range
	All (70)	MBI (33)		
STAI trait total	36.22 (0.85)	37.11 (1.36)	35.42 (1.10)	23–52
BDI total	9.91 (0.88)	10.62 (1.45)	9.17 (1.08)	0–32
DASS total	12.96 (1.26)	13.47 (2.00)	12.69 (1.64)	0–47
DASS depression	4.36 (0.50)	4.58 (0.86)	4.24 (0.58)	0–17
DASS anxiety	3.40 (0.42)	3.42 (0.55)	3.39 (0.66)	0–16
DASS stress	5.20 (0.51)	5.47 (0.81)	5.06 (0.65)	0–17
CAMM total	27.12 (0.90)	27.12 (1.34)	27.11 (1.24)	3–38
EAQ differentiating emotions	16.62 (0.36)	16.33 (0.59)	16.89 (0.44)	9–21
EAQ bodily awareness	9.45 (0.30)	9.45 (0.45)	9.44 (0.40)	5–14
ERQ cognitive reappraisal	16.84 (0.63)	15.79 (0.99)	17.81 (0.79)	6–27
ERQ expressive suppression	10.19 (0.45)	9.21 (0.66)	11.08 (0.59)	4–18
MASC total	32.26 (1.79)	32.45 (2.75)	32.08 (2.37)	8–75
MASC physical symptoms	8.11 (0.72)	8.34 (1.12)	7.89 (0.93)	0–27
MASC social anxiety	9.33 (0.72)	9.36 (1.08)	9.31 (0.96)	0–26
MASC self-harm	11.09 (0.56)	10.77 (0.84)	11.39 (0.76)	0–24
MASC separation anxiety	3.73 (0.36)	3.98 (0.56)	3.50 (0.45)	0–11
S&D total	10.71 (0.58)	10.79 (1.00)	10.54 (0.65)	1–23
S&D emotional symptoms	3.33 (0.30)	3.55 (0.46)	3.11 (0.41)	0–10
S&D conduct problems	2.09 (0.19)	2.03 (0.28)	2.11 (0.26)	0–6
S&D hyperactivity inattention	3.87 (0.29)	3.97 (0.43)	3.72 (0.40)	0–9
S&D peer problem	1.43 (0.18)	1.24 (0.25)	1.60 (0.27)	0–5
S&D prosocial behavior	8.54 (0.19)	8.33 (0.29)	8.74 (0.26)	4–10
PANAS positive	28.16 (0.81)	27.67 (1.16)	28.61 (1.15)	13–45
PANAS negative	14.39 (0.61)	14.64 (0.81)	14.17 (0.90)	10–36
WHO-5 total	15.55 (0.47)	15.42 (0.75)	15.67 (0.59)	4–23

**Table 3:** Group × visit interaction tests and V0–V2 contrasts within groups.

Outcome	Group × visit		BF	V0–V2: MBI		*t* (69)	*p*	BF	V0–V2: waiting		*t* (69)	*p*	BF
	*F* (1.69)	*p*		*D*	95% CI				*D*	95% CI			
STAIT	0.239	0.626	0.21	1.58	−0.46, 3.63	1.54	0.127	0.41	0.89	−1.07, 2.85	0.90	0.369	0.20
DASS total	0.015	0.904	0.16	1.74	−0.61, 4.09	1.47	0.145	0.32	1.94	−0.31, 4.19	1.72	0.091	0.46
MASC total	0.572	0.452	0.21	1.87	−1.45, 5.19	1.12	0.265	0.22	3.61	0.43, 6.79	2.27	0.027	1.34
WHO-5	0.086	0.771	0.25	0.09	−1.38, 1.56	0.12	0.902	0.18	0.39	0.55, −1.02	0.55	0.583	0.19
BDI	0.051	0.823	0.18	0.25	−1.92, 2.43	0.23	0.816	0.13	0.59	−1.49, 2.67	0.57	0.571	0.15
S&D total	0.000	0.997	0.20	0.37	−1.08, 1.83	0.51	0.613	0.13	0.38	−1.02, 1.77	0.54	0.593	0.16
PANAS positive	0.009	0.924	0.24	0.79	−1.55, 3.13	0.67	0.504	0.22	0.94	−1.30, 3.19	0.84	0.404	0.23
PANAS negative	0.014	0.906	0.26	−0.39	−2.48, 1.69	−0.38	0.708	0.21	−0.22	−2.22, 1.78	−0.22	0.825	0.19
CAMM total	0.029	0.864	0.19	0.82	−1.18, 2.81	0.82	0.416	0.18	1.06	−0.85, 2.97	1.10	0.274	0.22
ERQ CR	0.238	0.627	0.27	−0.65	−2.59, 1.28	−0.68	0.502	0.22	0.00	−1.85, 1.85	0.00	1.000	0.18
ERQ ES	0.771	0.383	0.30	−1.18	−2.44, 0.07	−1.88	0.065	0.78	−0.42	−1.61, 0.79	−0.69	0.492	0.21
EAQ BA	1.020	0.316	0.29	0.18	−0.47, 0.84	0.55	0.582	0.15	−0.28	−0.91, 0.35	−0.88	0.381	0.18
EAQ DE	1.032	0.313	0.35	−0.18	−1.13, 0.76	−0.38	0.702	0.17	−0.85	−1.77, 0.06	−1.86	0.068	0.76

## Data Availability

The data are available upon request through corresponding author.

## Endnotes

^1^Variables were transformed into residual scores that were obtained from a multilevel regression with visit as a fixed effect and subject as a random intercept. Residuals were calculated according to the procedure recommended by Fitzmaurice, Laird, and Ware.

^2^In the event influential cases and/or nonnormal residuals were found to be problematic, a nonparametric permutation repeated measures ANOVA was planned as a substitute.

## Supporting Information

Supporting Information 1 CONSORT_checklist: This document serves as a checklist of the CONSORT criteria for reporting clinical trials in accordance with publication guidelines.

Supporting Information 2 Supporting information contained in the file MTS_ClinicalPaper_Supp_Final.docx includes additional analysis results, which includes (a) Figure S1. Intervention effect graphs for the outcomes not plotted in the main manuscript. (b) Table S1. Intervention effects excluding participants with a chance to be randomized to a zoom intervention. (c) Table S2. Intervention effects excluding participants who received a zoom intervention. (d) Table S3. Follow-up moderator and confounder analyses of the intervention effects.

## References

[1] Spear L. P. (2013). Adolescent Neurodevelopment. *Journal of Adolescent Health*.

[2] Romeo R. D. (2010). Adolescence: A Central Event in Shaping Stress Reactivity. *Developmental Psychobiology*.

[3] Blakemore S.-J. (2019). Adolescence and Mental Health. *The Lancet*.

[4] Tottenham N., Galván A. (2016). Stress and the Adolescent Brain: Amygdala-Prefrontal Cortex Circuitry and Ventral Striatum as Developmental Targets. *Neuroscience & Biobehavioral Reviews*.

[5] Casey B. J., Heller A. S., Gee D. G., Cohen A. O. (2019). Development of the Emotional Brain. *Neuroscience Letters*.

[6] Monroe S. M., Harkness K. L. (2005). Life Stress, the “Kindling” Hypothesis, and the Recurrence of Depression: Considerations From a Life Stress Perspective. *Psychological Review*.

[7] Paus T., Keshavan M., Giedd J. N. (2008). Why Do Many Psychiatric Disorders Emerge During Adolescence?. *Nature Reviews Neuroscience*.

[8] Roberts A. G., Lopez-Duran N. L. (2019). Developmental Influences on Stress Response Systems: Implications for Psychopathology Vulnerability in Adolescence. *Comprehensive Psychiatry*.

[9] Solmi M., Radua J., Olivola M. (2022). Age at Onset of Mental Disorders Worldwide: Large-Scale Meta-Analysis of 192 Epidemiological Studies. *Molecular Psychiatry*.

[10] Beauchaine T. P. (2015). Future Directions in Emotion Dysregulation and Youth Psychopathology. *Journal of Clinical Child & Adolescent Psychology*.

[11] Cracco E., Goossens L., Braet C. (2017). Emotion Regulation Across Childhood and Adolescence: Evidence for a Maladaptive Shift in Adolescence. *European Child & Adolescent Psychiatry*.

[12] Fernandez K. C., Jazaieri H., Gross J. J. (2016). Emotion Regulation: A Transdiagnostic Perspective on a New RDoC Domain. *Cognitive Therapy and Research*.

[13] Beauchaine T. P., Cicchetti D. (2019). Emotion Dysregulation and Emerging Psychopathology: A Transdiagnostic, Transdisciplinary Perspective. *Development and Psychopathology*.

[14] Kebets V., Favre P., Houenou J. (2021). Fronto-Limbic Neural Variability as a Transdiagnostic Correlate of Emotion Dysregulation. *Translational Psychiatry*.

[15] Duffy A., Horrocks J., Doucette S., Keown-Stoneman C., McCloskey S., Grof P. (2014). The Developmental Trajectory of Bipolar Disorder. *British Journal of Psychiatry*.

[16] Christiansen H., Hirsch O., Albrecht B., Chavanon M.-L. (2019). Attention-Deficit/Hyperactivity Disorder (ADHD) and Emotion Regulation Over the Life Span. *Current Psychiatry Reports*.

[17] Murray R. J., Gentsch K., Pham E. (2022). Identifying Disease-Specific Neural Reactivity to Psychosocial Stress in Borderline Personality Disorder. *Biological Psychiatry: Cognitive Neuroscience and Neuroimaging*.

[18] Hickie I. B., Scott E. M., Hermens D. F. (2013). Applying Clinical Staging to Young People Who Present for Mental Health Care. *Early Intervention in Psychiatry*.

[19] McGorry P., Nelson B. (2016). Why We Need a Transdiagnostic Staging Approach to Emerging Psychopathology, Early Diagnosis, and Treatment. *JAMA Psychiatry*.

[20] Shah J. L., Scott J., McGorry P. D. (2020). Transdiagnostic Clinical Staging in Youth Mental Health: A First International Consensus Statement. *World Psychiatry*.

[21] Shah J. L., Jones N., van Os J., McGorry P. D., Gülöksüz S. (2022). Early Intervention Service Systems for Youth Mental Health: Integrating Pluripotentiality, Clinical Staging, and Transdiagnostic Lessons From Early Psychosis. *The Lancet Psychiatry*.

[22] Ratheesh A., Cotton S. M., Davey C. G. (2017). Ethical Considerations in Preventive Interventions for Bipolar Disorder. *Early Intervention in Psychiatry*.

[23] Kalisch R., Baker D. G., Basten U. (2017). The Resilience Framework as a Strategy to Combat Stress-Related Disorders. *Nature Human Behaviour*.

[24] Dray J. (2021). Child and Adolescent Mental Health and Resilience-Focussed Interventions: A Conceptual Analysis to Inform Future Research. *International Journal of Environmental Research and Public Health*.

[25] Mesman E., Vreeker A., Hillegers M. (2021). Resilience and Mental Health in Children and Adolescents: An Update of the Recent Literature and Future Directions. *Current Opinion in Psychiatry*.

[26] Konaszewski K., Niesiobędzka M., Surzykiewicz J. (2021). Resilience and Mental Health Among Juveniles: Role of Strategies for Coping With Stress. *Health and Quality of Life Outcomes*.

[27] Kołodziejska M., Paliński M. (2022). ’Train Your Mind for a Healthy Life’. The mMedicalization of mMediatized mMindfulness in the West. *Current Psychology*.

[28] Kabat-Zinn J. (2005). *Coming to Our Senses: Healing Ourselves and the World Through Mindfulness*.

[29] Alsubaie M., Abbott R., Dunn B. (2017). Mechanisms of Action in Mindfulness-Based Cognitive Therapy (MBCT) and Mindfulness-Based Stress Reduction (MBSR) in People with Physical and/or Psychological Conditions: A Systematic Review. *Clinical Psychology Review*.

[30] Allen J. G., Romate J., Rajkumar E. (2021). Mindfulness-Based Positive Psychology Interventions: A Systematic Review. *BMC Psychology*.

[31] Zhang D., Lee E. K. P., Mak E. C. W., Ho C. Y., Wong S. Y. S. (2021). Mindfulness-Based Interventions: An Overall Review. *British Medical Bulletin*.

[32] Gu J., Strauss C., Bond R., Cavanagh K. (2015). How Do Mindfulness-Based Cognitive Therapy and Mindfulness-Based Stress Reduction Improve Mental Health and Wellbeing? A Systematic Review and Meta-Analysis of Mediation Studies. *Clinical Psychology Review*.

[33] Galante J., Friedrich C., Dalgleish T. (2023). Systematic Review and Individual Participant Data Meta-Analysis of Randomized Controlled Trials Assessing Mindfulness-Based Programs for Mental Health Promotion. *Nature Mental Health*.

[34] Hoge E. A., Bui E., Mete M., Dutton M. A., Baker A. W., Simon N. M. (2023). Mindfulness-Based Stress Reduction Vs Escitalopram for the Treatment of Adults With Anxiety Disorders. *JAMA Psychiatry*.

[35] Perry-Parrish C., Copeland-Linder N., Webb L., Shields A. H., Sibinga E. M. (2016). Improving Self-Regulation in Adolescents: Current Evidence for the Role of Mindfulness-Based Cognitive Therapy. *Adolescent Health, Medicine and Therapeutics*.

[36] Vo D. X., Doyle J., Christie D. (2014). Mindfulness and Adolescence: A Clinical Review of Recent Mindfulness-Based Studies in Clinical and Nonclinical Adolescent Populations. *Adolescent Medicine: State of the Art Reviews*.

[37] Zoogman S., Goldberg S. B., Hoyt W. T., Miller L. (2015). Mindfulness Interventions With Youth: A Meta-Analysis. *Mindfulness*.

[38] Odgers K., Dargue N., Creswell C., Jones M. P., Hudson J. L. (2020). The Limited Effect of Mindfulness-Based Interventions on Anxiety in Children and Adolescents: A Meta-Analysis. *Clinical Child and Family Psychology Review*.

[39] Dunning D., Tudor K., Radley L. (2022). Do Mindfulness-Based Programmes Improve the Cognitive Skills, Behaviour and Mental Health of Children and Adolescents? An Updated Meta-Analysis of Randomised Controlled Trials. *Evidence Based Mental Health*.

[40] Chi X., Bo A., Liu T., Zhang P., Chi I. (2018). Effects of Mindfulness-Based Stress Reduction on Depression in Adolescents and Young Adults: A Systematic Review and Meta-Analysis. *Frontiers in Psychology*.

[41] Scafuto F., Ghiroldi S., Montecucco N. F. (2024). Promoting Well-Being in Early Adolescents Through Mindfulness: A Cluster Randomized Controlled Trial. *Journal of Adolescence*.

[42] Kuyken W., Ball S., Crane C. (2022). Effectiveness and Cost-Effectiveness of Universal School-Based Mindfulness Training Compared With Normal School Provision in Reducing Risk of Mental Health Problems and Promoting Well-Being in Adolescence: The MYRIAD Cluster Randomised Controlled Trial. *Evidence Based Mental Health*.

[43] Schuman-Olivier Z., Trombka M., Lovas D. A. (2020). Mindfulness and Behavior Change. *Harvard Review of Psychiatry*.

[44] Piguet C., Klauser P., Celen Z., James Murray R., Magnus Smith M., Merglen A. (2021). Randomized Controlled Trial of a Mindfulness-Based Intervention in Adolescents From the General Population: The Mindfulteen Neuroimaging Study Protocol. *Early Intervention in Psychiatry*.

[45] Siffredi V., Liverani M. C., Hüppi P. S. (2021). The Effect of a Mindfulness-Based Intervention on Executive, Behavioural and Socio-Emotional Competencies in Very Preterm Young Adolescents. *Scientific Reports*.

[46] Inserm (2002). *Kiddie-Sads: Version Vie Entière* (*K-SADS-P/L*) *6-18 Ans*.

[47] Turgeon L., Chartrand É. (2003). Psychometric Properties Of the French Canadian Version of the State-Trait Anxiety Inventory For Children. *Educational and Psychological Measurement*.

[48] Giacomo F. D., Strippoli M.-P. F., Castelao E. (2023). Risk Factors for Mood Disorders Among Offspring of Parents With Bipolar Disorder: Findings From a Discordant-Sibling Study. *Psychiatry Research*.

[49] March J. S., Parker J. D., Sullivan K., Stallings P., Conners C. K. (1997). The Multidimensional Anxiety Scale for Children (MASC): Factor Structure, Reliability, and Validity. *Journal of the American Academy of Child & Adolescent Psychiatry*.

[50] Nahaboo S. (2015). Validation of the French Depression Anxiety Stress Scales (DASS-21) and Predictors of Depression in an Adolescent Mauritian Population.

[51] Beck A. T., Steer R. A., Brown G. K. (1996). *Manual for the Beck Depression Inventory*.

[52] Goodman R. (1997). The Strengths and Difficulties Questionnaire: A Research Note. *Journal of Child Psychology and Psychiatry, and Allied Disciplines*.

[53] Dion J., Paquette L., Daigneault I., Godbout N., Hébert M. (2018). Validation of the French Version of the Child and Adolescent Mindfulness Measure (CAMM) Among Samples of French and Indigenous Youth. *Mindfulness*.

[54] Gaudreau P., Sanchez X., Blondin J.-P. (2006). Positive and Negative Affective States in a Performance-Related Setting: Testing the Factorial Structure of the Panas Across Two Samples of French-Canadian Participants. *European Journal of Psychological Assessment*.

[55] Gullone E., Taffe J. (2012). The Emotion Regulation Questionnaire for Children and Adolescents (ERQ-CA): A Psychometric Evaluation. *Psychological Assessment*.

[56] Lahaye M., Luminet O., Van Broeck N., Bodart E., Mikolajczak M. (2010). Psychometric Properties of the Emotion Awareness Questionnaire for Children in a French-Speaking Population. *Journal of Personality Assessment*.

[57] Benjamin D. J., Berger J. O., Johannesson M. (2018). Redefine Statistical Significance. *Nature Human Behaviour*.

[58] R Core Team (2022). R: A Language and Environment for Statistical Computing. https://www.R-project.org/.

[59] Bates D., Mächler M., Bolker B., Walker S. (2015). Fitting Linear Mixed-Effects Models Using lme4. *Journal of Statistical Software*.

[60] Kuznetsova A., Brockhoff P. B., Christensen R. H. B. (2017). lmerTest Package: Tests in Linear Mixed Effects Models. *Journal of Statistical Software*.

[61] Hartig F., Lohse L. (2022). *DHARMa: Residual Diagnostics for Hierarchical* (*Multi-Level/Mixed*) *Regression Models* (Version 0.4.6) [Computer Software]. https://CRAN.R-project.org/package=DHARMa.

[62] Bürkner P.-C. (2018). Advanced Bayesian Multilevel Modeling With the R Package Brms. *The R Journal*.

[63] Makowski D., Ben-Shachar M., Lüdecke D. (2019). bayestestR: Describing Effects and Their Uncertainty, Existence and Significance Within the Bayesian Framework. *Journal of Open Source Software*.

[64] Breheny P., Burchett W. (2017). Visualization of Regression Models Using visreg. *The R Journal*.

[65] Taiyun (2023). *Taiyun/Corrplot* [R]. https://github.com/taiyun/corrplot.

[66] Johnson C., Taylor A., Dray J., Dunning D. (2024). You Can Lead an Adolescent to Mindfulness, But You Can’t Make Them Mindful. *Mindfulness*.

[67] Allan N. P., Capron D. W., Lejuez C. W., Reynolds E. K., MacPherson L., Schmidt N. B. (2014). Developmental Trajectories of Anxiety Symptoms in Early Adolescence: The Influence of Anxiety Sensitivity. *Journal of Abnormal Child Psychology*.

[68] Theurel A., Gentaz E. (2018). The Regulation of Emotions in Adolescents: Age Differences and Emotion-Specific Patterns. *PLoS ONE*.

[69] McLaughlin K. A., King K. (2015). Developmental Trajectories of Anxiety and Depression in Early Adolescence. *Journal of Abnormal Child Psychology*.

[70] Holman C., Magnus Smith M., Liverani M. C. A Qualitative Study of Adolescents’ Perception and Reported Effects of a Mindfulness-Based Intervention.

[71] Johnson C., Wade T. (2019). Piloting a More Intensive 8-Week Mindfulness Programme in Early- and Mid-Adolescent School Students. *Early Intervention in Psychiatry*.

[72] Robe A., Dobrean A. (2023). The Effectiveness of a Single Session of Mindfulness-Based Cognitive Training on Cardiac Vagal Control and Core Symptoms in Children and Adolescents With Attention-Deficit/Hyperactivity Disorder (ADHD): A Preliminary Randomized Controlled Trial. *European Child & Adolescent Psychiatry*.

[73] Strohmaier S., Bailey N. W. (2023). Do Not Keep Calm and Carry On: School-Based Mindfulness Programmes Should Test Making Mindfulness Practice Available in the School Day. *Mindfulness*.

[74] Schlosser M., Sparby T., Vörös S., Jones R., Marchant N. L. (2019). Unpleasant Meditation-Related Experiences in Regular Meditators: Prevalence, Predictors, and Conceptual Considerations. *PLoS ONE*.

[75] Goldberg S. B., Lam S. U., Britton W. B., Davidson R. J. (2022). Prevalence of Meditation-Related Adverse Effects in a Population-Based Sample in the United States. *Psychotherapy Research: Journal of the Society for Psychotherapy Research*.

[76] Britton W. B. (2019). Can Mindfulness Be Too Much of a Good Thing? The Value of a Middle Way. *Current Opinion in Psychology*.

[77] Gotink R. A., Vernooij M. W., Ikram M. A. (2018). Meditation and Yoga Practice Are Associated With Smaller Right Amygdala Volume: The Rotterdam Study. *Brain Imaging and Behavior*.

[78] Hoge E. A., Bui E., Marques L. (2013). Randomized Controlled Trial of Mindfulness Meditation for Generalized Anxiety Disorder: Effects on Anxiety and Stress Reactivity. *The Journal of Clinical Psychiatry*.

[79] Galante J., Friedrich C., Dawson A. F. (2021). Mindfulness-Based Programmes for Mental Health Promotion in Adults in Nonclinical Settings: A Systematic Review and Meta-Analysis of Randomised Controlled Trials. *PLoS Medicine*.

[80] Taddeo D., Egedy M., Frappier J.-Y. (2008). Adherence to Treatment in Adolescents. *Paediatrics & Child Health*.

[81] McCartney M., Nevitt S., Lloyd A., Hill R., White R., Duarte R. (2021). Mindfulness-Based Cognitive Therapy for Prevention and Time to Depressive Relapse: Systematic Review and Network Meta-Analysis. *Acta Psychiatrica Scandinavica*.

[82] Jones E. A. K., Mitra A. K., Bhuiyan A. R. (2021). Impact of COVID-19 on Mental Health in Adolescents: A Systematic Review. *International Journal of Environmental Research and Public Health*.

[83] Yuan Y. (2021). Mindfulness Training on the Resilience of Adolescents Under the COVID-19 Epidemic: A Latent Growth Curve Analysis. *Personality and Individual Differences*.

[84] Li Y., Ma X., Feng C., Wang Y. (2022). Parental Psychological Control and Adolescents Depression during the COVID-19 Pandemic: The Mediating and Moderating Effect of Self-Concept Clarity and Mindfulness. *Current Psychology (New Brunswick, N.J.)*.

[85] Johnson C., Burke C., Brinkman S., Wade T. (2016). Effectiveness of a School-Based Mindfulness Program for Transdiagnostic Prevention in Young Adolescents. *Behaviour Research and Therapy*.

[86] Volanen S.-M., Lassander M., Hankonen N. (2020). Healthy Learning Mind—Effectiveness of a Mindfulness Program on Mental Health Compared to a Relaxation Program and Teaching as Usual in Schools: A Cluster-Randomised Controlled Trial. *Journal of Affective Disorders*.

[87] Buric I., Farias M., Driessen J. M. A., Brazil I. A. (2022). Individual Differences in Meditation Interventions: A Meta-Analytic Study. *British Journal of Health Psychology*.

[88] Galante J., Friedrich C., Dalgleish T., White I. R., Jones P. B. (2022). Mindfulness-Based Programmes for Mental Health Promotion in Adults in Non-Clinical Settings: Protocol of an Individual Participant Data Meta-Analysis of Randomised Controlled Trials. *BMJ Open*.

