# Supplementary material for: Impact of a Mindfulness-Based Intervention on Symptoms and Emotion Regulation Strategies in Young Adolescents From the General Population: A Randomized Controlled Trial
Source: Depress Anxiety. 2025 Jun 10;2025:2679049. doi: 10.1155/da/2679049 (PMC12173555; doi:10.1155/da/2679049)
Supplement: Supporting Information 2 — Supporting information contained in the file MTS_ClinicalPaper_Supp_Final.docx includes additional analysis results, which includes (a) Figure S1. Intervention effect graphs for the outcomes not plotted in the main manuscript. (b) Table S1. Intervention effects excluding participants with a chance to be randomized to a zoom intervention. (c) Table S2. Intervention effects excluding participants who received a zoom intervention. (d) Table S3. Follow-up moderator and confounder analyses of the intervention effects. [file 2679049.f2.docx]

Impact of a mindfulness-based intervention on symptoms and emotion regulation strategies in young adolescents from the general population: a randomized controlled trial: **supplementary material**

Camille Piguet^1,2^, Zeynep Celen^2^, Ben Meuleman^2,3^, Zoé Schilliger^4,5^, Mariana Magnus Smith^1,2^, Erik Mendola^1^, Eleonore Pham^2^, Sondes Jouabli^2^, Vladimira Ivanova^2^, Ryan Murray,^2,5^ Paul Klauser^4,5^*, Arnaud Merglen^1^*

^1^General Pediatrics Division, Women, Child and Adolescent Department, Geneva University Hospital, Switzerland

^2^Psychiatric Department, Faculty of Medicine, University of Geneva, Switzerland

^3^Swiss Center for Affective Sciences, University of Geneva, Switzerland.

^4^Center for Psychiatric Neuroscience, Department of Psychiatry, Lausanne University Hospital and the University of Lausanne, Lausanne, Switzerland

^5^ Service of Child and Adolescent Psychiatry, Department of Psychiatry, Lausanne University

Hospital and the University of Lausanne, Lausanne, Switzerland

Corresponding Author: Camille Piguet

*these authors contributed equally to the work

Email: camille.piguet@unige.ch

ORCID: [0000-0003-4317-0918](https://orcid.org/0000-0003-4317-0918)


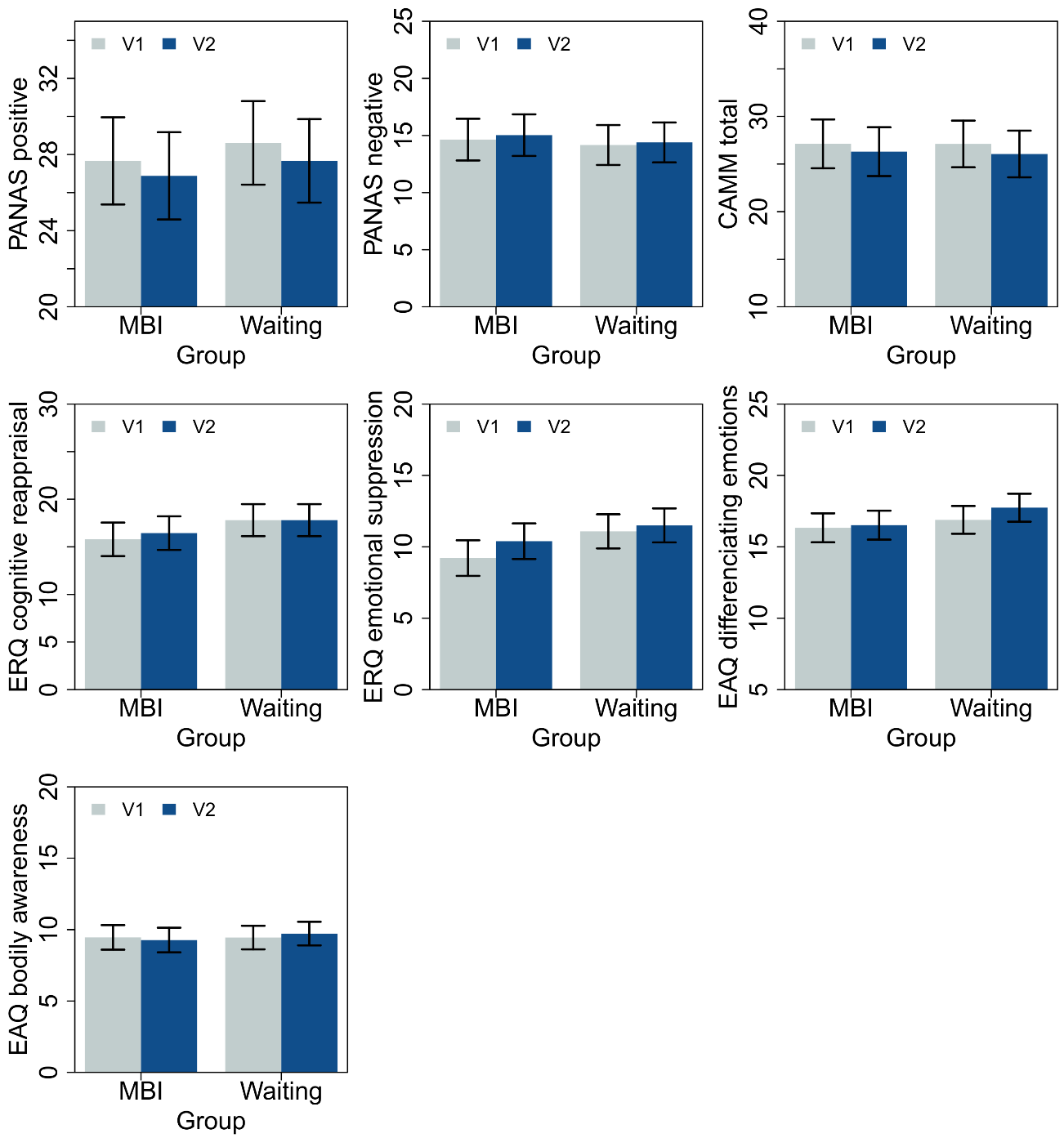


Figure S1. Group × Visit estimated means and standard errors for CAMM total score and PANAS, ERQ, and EAQ sub-scores

Table S1. Group × Visit interaction tests and V0-V2 contrasts within groups, excluding participants who had the chance to be randomized to the Zoom intervention.

|  | **Group × Visit** | |  | **V0-V2: Early** | |  |  |  | **V0-V2: Waiting** | |  |  |  |
| --- | --- | --- | --- | --- | --- | --- | --- | --- | --- | --- | --- | --- | --- |
| **Outcome** | ***F*(1,50)** | ***p*** | ***BF*** | ***D*** | **95%CI** | ***t*(50)** | ***p*** | ***BF*** | ***D*** | **95%CI** | ***t*(50)** | **p** | **BF** |
| *STAIT* | 0.160 | 0.690 | 0.19 | 1.25 | -0.91,3.41 | 1.16 | 0.249 | 0.25 | 0.65 | -1.42,2.73 | 0.63 | 0.528 | 0.16 |
| *DASS total* | 0.006 | 0.940 | 0.16 | 0.86 | -1.69,3.41 | 0.68 | 0.501 | 0.14 | 0.99 | -1.46,3.45 | 0.81 | 0.419 | 0.15 |
| *MASC total* | 0.476 | 0.493 | 0.19 | 1.96 | -1.61,5.52 | 1.10 | 0.276 | 0.20 | 3.65 | 0.23,7.08 | 2.14 | 0.037 | 0.92 |
| *WHO-5* | 0.149 | 0.700 | 0.27 | 0.88 | -0.82,2.57 | 1.04 | 0.303 | 0.31 | 0.42 | -1.20,2.05 | 0.52 | 0.603 | 0.22 |
| *BDI* | 0.157 | 0.694 | 0.19 | -0.61 | -3.08,1.87 | -0.49 | 0.623 | 0.15 | 0.07 | -2.31,2.45 | 0.06 | 0.954 | 0.12 |
| *S&D total* | 0.000 | 0.986 | 0.18 | -0.04 | -1.39,1.30 | -0.06 | 0.951 | 0.13 | -0.06 | -1.35,1.23 | -0.09 | 0.929 | 0.13 |
| *PANAS positive* | 0.045 | 0.832 | 0.28 | 0.71 | -2.07,3.49 | 0.51 | 0.610 | 0.23 | 1.12 | -1.55,3.78 | 0.84 | 0.405 | 0.27 |
| *PANAS negative* | 0.045 | 0.833 | 0.28 | -0.17 | -2.36,2.03 | -0.15 | 0.879 | 0.20 | 0.15 | -1.95,2.26 | 0.15 | 0.884 | 0.20 |
| *CAMM total* | 0.107 | 0.745 | 0.20 | 0.54 | -1.66,2.74 | 0.49 | 0.623 | 0.15 | 1.04 | -1.08,3.15 | 0.99 | 0.329 | 0.20 |
| *ERQ CR* | 0.001 | 0.975 | 0.29 | 0.13 | -2.16,2.41 | 0.11 | 0.912 | 0.21 | 0.08 | -2.12,2.27 | 0.07 | 0.944 | 0.20 |
| *ERQ ES* | 2.213 | 0.143 | 0.65 | -1.17 | -2.64,0.31 | -1.59 | 0.117 | 0.52 | 0.35 | -1.07,1.76 | 0.49 | 0.625 | 0.20 |
| EAQ BA | 0.394 | 0.533 | 0.27 | 0.00 | -0.80,0.80 | 0.00 | 1.000 | 0.16 | -0.35 | -1.11,0.42 | -0.91 | 0.369 | 0.23 |
| EAQ DE | 0.057 | 0.813 | 0.23 | -0.75 | -1.80,0.30 | -1.43 | 0.159 | 0.41 | -0.92 | -1.94,0.08 | -1.83 | 0.073 | 0.78 |

Table S2. Group × Visit interaction tests and V0-V2 contrasts within groups, excluding participants who received a Zoom intervention.

|  | **Group × Visit** | |  | **V0-V2: Early** | |  |  |  | **V0-V2: Waiting** | |  |  |  |
| --- | --- | --- | --- | --- | --- | --- | --- | --- | --- | --- | --- | --- | --- |
| **Outcome** | ***F*(1,64)** | ***p*** | ***BF*** | ***D*** | **95%CI** | ***t*(64)** | ***p*** | ***BF*** | ***D*** | **95%CI** | ***t*(64)** | **p** | **BF** |
| *STAIT* | 0.683 | 0.411 | 0.25 | 2.09 | -0.08,4.25 | 1.92 | 0.059 | 0.77 | 0.89 | -1.02,2.80 | 0.93 | 0.356 | 0.20 |
| *DASS total* | 0.150 | 0.700 | 0.17 | 1.27 | -1.29,3.83 | 0.99 | 0.323 | 0.20 | 1.94 | -0.32,4.19 | 1.71 | 0.091 | 0.43 |
| *MASC total* | 0.315 | 0.576 | 0.19 | 2.25 | -1.39,5.89 | 1.23 | 0.222 | 0.25 | 3.61 | 0.40,6.82 | 2.25 | 0.028 | 1.10 |
| *WHO-5* | 0.030 | 0.864 | 0.24 | 0.57 | -1.02,2.16 | 0.71 | 0.475 | 0.23 | 0.39 | -1.01,1.79 | 0.55 | 0.581 | 0.19 |
| *BDI* | 0.304 | 0.583 | 0.20 | -0.27 | -2.62,2.08 | -0.23 | 0.818 | 0.14 | 0.59 | -1.48,2.67 | 0.57 | 0.569 | 0.14 |
| *S&D total* | 0.037 | 0.848 | 0.21 | 0.57 | -0.95,2.10 | 0.75 | 0.457 | 0.20 | 0.38 | -0.97,1.72 | 0.56 | 0.580 | 0.16 |
| *PANAS positive* | 0.241 | 0.907 | 0.27 | 0.14 | -2.30,2.59 | 0.12 | 0.907 | 0.18 | 0.94 | -1.21,3.10 | 0.87 | 0.385 | 0.23 |
| *PANAS negative* | 0.003 | 0.955 | 0.26 | -0.14 | -2.26,1.97 | -0.13 | 0.893 | 0.20 | -0.22 | -2.09,1.64 | -0.24 | 0.812 | 0.18 |
| *CAMM total* | 0.047 | 0.829 | 0.19 | 0.75 | -1.36,2.86 | 0.71 | 0.480 | 0.17 | 1.06 | -0.81,2.92 | 1.13 | 0.262 | 0.23 |
| *ERQ CR* | 0.188 | 0.666 | 0.29 | -0.63 | -2.80,1.54 | -0.58 | 0.565 | 0.23 | 0.00 | -1.92,1.92 | 0.00 | 1.000 | 0.18 |
| *ERQ ES* | 1.718 | 0.194 | 0.46 | -1.61 | -2.97,-0.25 | -2.36 | 0.021 | 2.00 | -0.42 | -1.62,0.78 | -0.69 | 0.490 | 0.22 |
| EAQ BA | 0.345 | 0.558 | 0.21 | 0.00 | -0.71,0.71 | 0.00 | 1.000 | 0.14 | -0.28 | -0.90,0.35 | -0.89 | 0.377 | 0.18 |
| EAQ DE | 0.718 | 0.400 | 0.31 | -0.29 | -1.28,0.71 | -0.57 | 0.567 | 0.19 | -0.85 | -1.74,0.04 | -1.91 | 0.060 | 0.83 |

Table S3. Follow-up moderator and confounder analyses of the Group x Visit effect. P-values are reported for the three-way interaction (moderators), or the two-way interaction (confounders).

|  | **Moderator effects** *(Group × Visit × Moderator)* | | | **Confounder effects**  *(Group × Visit + Confounder)* | |
| --- | --- | --- | --- | --- | --- |
| **Outcome** | **Anxiety strata** | **GAD** | **Depression** | **Time (age)** | **Time (month)** |
| STAIT | 0.0464 | 0.7335 | 0.0019 | 0.7127 | 0.4569 |
| DASS total | 0.0569 | 0.7220 | 0.0047 | 0.7815 | 0.8607 |
| MASC total | 0.7707 | 0.9745 | 0.6832 | 0.3778 | 0.5191 |
| WHO-5 | 0.6491 | 0.3314 | 0.3868 | 0.8813 | 0.7402 |
| BDI | 0.0979 | 0.4950 | 0.0038 | 0.7227 | 0.9277 |
| S&D total | 0.4217 | 0.4252 | 0.0170 | 0.8591 | 0.8885 |
| PANAS positive | 0.7887 | 0.1226 | 0.6815 | 0.7325 | 0.8077 |
| PANAS negative | 0.1187 | 0.5307 | 0.1501 | 0.7327 | 0.2447 |
| CAMM total | 0.7232 | 0.6881 | 0.2324 | 0.9542 | 0.8387 |
| ERQ CR | 0.9201 | 0.4206 | 0.2402 | 0.5280 | 0.0396 |
| ERQ ES | 0.2363 | 0.9404 | 0.6085 | 0.2906 | 0.0761 |
| EAQ BA | 0.9355 | 0.5861 | 0.0273 | 0.3510 | 0.2031 |
| EAQ DE | 0.4745 | 0.4144 | 0.0046 | 0.3893 | 0.3179 |
